# Comprehensive evaluation of structural variant genotyping methods based on long-read sequencing data

**DOI:** 10.1186/s12864-022-08548-y

**Published:** 2022-04-23

**Authors:** Xiaoke Duan, Mingpei Pan, Shaohua Fan

**Affiliations:** 1 State Key Laboratory of Genetic Engineering, Human Phenome Institute, Zhangjiang Fudan International Innovation Center, Fudan University, Shanghai, 200438 China; 2 MOE Key Laboratory of Contemporary Anthropology, Department of Anthropology and Human Genetics, School of Life Sciences, Fudan University, Shanghai, 200433 China

**Keywords:** Long-read sequencing, SV genotyping, F1 score, Performance evaluation

## Abstract

**Background:**

Structural variants (SVs) play a crucial role in gene regulation, trait association, and disease in humans. SV genotyping has been extensively applied in genomics research and clinical diagnosis. Although a growing number of SV genotyping methods for long reads have been developed, a comprehensive performance assessment of these methods has yet to be done.

**Results:**

Based on one simulated and three real SV datasets, we performed an in-depth evaluation of five SV genotyping methods, including cuteSV, LRcaller, Sniffles, SVJedi, and VaPoR. The results show that for insertions and deletions, cuteSV and LRcaller have similar F1 scores (cuteSV, insertions: 0.69–0.90, deletions: 0.77–0.90 and LRcaller, insertions: 0.67–0.87, deletions: 0.74–0.91) and are superior to other methods. For duplications, inversions, and translocations, LRcaller yields the most accurate genotyping results (0.84, 0.68, and 0.47, respectively). When genotyping SVs located in tandem repeat region or with imprecise breakpoints, cuteSV (insertions and deletions) and LRcaller (duplications, inversions, and translocations) are better than other methods. In addition, we observed a decrease in F1 scores when the SV size increased. Finally, our analyses suggest that the F1 scores of these methods reach the point of diminishing returns at 20× depth of coverage.

**Conclusions:**

We present an in-depth benchmark study of long-read SV genotyping methods. Our results highlight the advantages and disadvantages of each genotyping method, which provide practical guidance for optimal application selection and prospective directions for tool improvement.

**Supplementary Information:**

The online version contains supplementary material available at 10.1186/s12864-022-08548-y.

## Background

Structural variants (SVs) are genomic alterations of at least 50 bp in size, including insertions (INSs), deletions (DELs), duplications (DUPs), inversions (INVs), and translocations (TRAs) [1]. Although the number of SVs (20–30 k) is less abundant than single-nucleotide variants (SNVs, 3–4 M), these can cause more than three times more base-pair differences among humans than SNVs [2]. Recent studies have demonstrated SVs play an important role in gene expression [3, 4], phenotypic diversity [5–9], monogenic and complex diseases [10–12] in humans.

The identification of SVs mainly includes two stages: discovery and genotyping [13]. Discovery refers to the de novo detection process of discordant signatures between the sequenced individual and the reference genome [13]. It aims to discover and characterize SVs at a genome-wide scale, including the type, size, and position of an SV [13]. Genotyping is the process of determining the presence and absence of variants in a given individual based on known and characterized SVs [13]. It is more targeted and simpler than the SV discovery stage [14, 15]. Genotyping has major application values in clinical diagnoses [15] and basic science studies [16–19]. For instance, focusing on known clinically relevant SVs, genotyping can directly examine the presence/absence of an SV in sequenced patient samples [15]. In pedigree analysis, genotyping can identify de novo SVs (those are in the offspring with disease conditions and are not present in the unaffected parents) and is widely used for the diagnosis of rare and complex genetic diseases [16, 17]. Genotyping across population-scale samples increases the recall under low coverages and provides the basis for genome-wide association studies [18, 19].

Numerous SV genotyping methods, which were based on short-read sequencing (SRS) data, have been developed in the past few years, including SVTyper [20], BayesTyper [21], Paragraph [22], vg [23], and Graphtyper2 [24]. However, previous studies have shown that these methods have serious drawbacks mainly owing to the limitations of SRS data (e.g., uneven coverage across the genome [25], failure to sequence highly repetitive region, and incapable of unambiguously mapping reads to the regions that are polymorphic or not unique due to short read length [26]). First, these methods have poor genotyping accuracy for SVs in tandem repeat (TR) regions. Their false discovery rates are at least 40% [27]. Second, these methods are limited to specific SV types. A prior study [15] evaluated five SV genotyping methods based on SRS data, but none of these can genotype INS. Third, customized VCF files or information are required (e.g., paragraph requires precise breakpoints of the targeted SVs [22]).

Platforms of Pacific Biosciences’ (PacBio) single-molecule real-time (SMRT) sequencing [28] and Oxford Nanopore Technologies’ (ONT) nanopore sequencing [29] dominate the long-read sequencing (LRS) market. PacBio sequencing technology uses a topologically circular DNA molecule template (known as SMRTbell) to integrate double-stranded DNA ranging from one to more than a hundred kilobases base pairs. The PacBio platform generates continuous long reads (CLR) (read N50: 5–60 kb; accuracy: 87–92%) or circular consensus sequencing (CCS) reads (read N50: 10–20 kb; accuracy: > 99%) [30]. ONT sequencing technology utilizes linear DNA molecules and infers sequence of bases based on ionic current fluctuations caused by a single-stranded DNA passing through biological nanopores. The ONT platform generates long (read N50: 10–60 kb; accuracy: 87–98%) or ultra-long (read N50: 100–200 kb; accuracy: 87–98%) reads [30]. With a read length > 10 kb and the ability to read through highly repetitive regions in the human genome, LRS technologies are revolutionizing the study of SVs [30–34]. A benchmark study from the Genome in a Bottle (GIAB) Consortium showed that methods using SRS data can only genotype 65% of deletions and 53% of insertions in tandem repeats when evaluating their benchmark SVs [35]. Another study showed that SVJedi using LRS data had a two-fold increase in genotyping accuracy than SVtyper, which is based on SRS data [14].

Although an increasing number of LRS-based genotyping methods have been published, the performance of these methods has not been comprehensively evaluated. In this study, we benchmarked five LRS-based SV genotyping methods, namely, cuteSV [36], LRcaller [32], Sniffles [37], SVJedi [14], and VaPoR [38] on both simulated and real LRS datasets. We present a comprehensive assessment of genotyping accuracy of these SV genotyping methods based on multiple different factors, including SV size, breakpoint located in TR regions, imprecise breakpoints, aligner, sequencing data type, and depth of coverage. Furthermore, we compare computational resource consumption. Our study highlights both the strengths and limitations of LRS-based SV genotyping methods to assist in the development of future SV genotyping methods and practical applications to genomic and clinical studies.

## Results

### Benchmark datasets

We collected one simulated and three real SV datasets (Table 1) in the present study. The simulated dataset was generated using VISOR [39]. The simulated SV set includes a total of 15,453 heterozygous (0/1) and homozygous non-reference (1/1) SVs with sizes ranging from 0.05 to 364 kb. We also simulated 30× PacBio CLR data using VISOR (see the “Methods” section for details).

**Table 1:** Summary of the SV sets

SV set	Total	INS	DEL	DUP	INV	TRA	SV size (kb)
Simulated data	15,453	7710	7290	167	72	214	0.05–364
HG002 Tier 1	12,745	7281	5464	NA	NA	NA	0.05–125
HG002 Tier 2	7001	4189	2812	NA	NA	NA	0.05–240
HG005	17,447	8867	8121	296	38	125	0.05–73

Total is the total number of SVs for each SV set. “NA”: data are not available

**Fig. 1:**
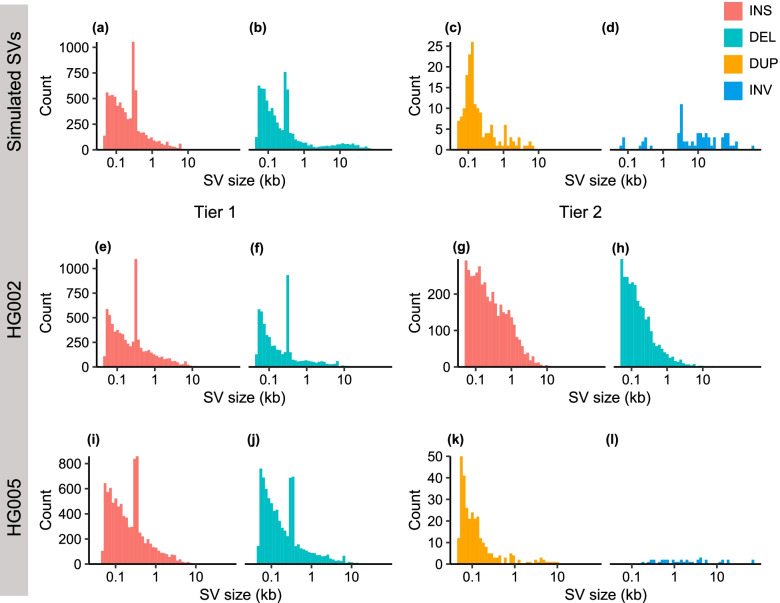
Size distribution of SV sets. **a-d** The simulated SV set using VISOR. **e, f **HG002 Tier 1 SV set. **g, h** HG002 Tier 2 SV set. **i-l** HG005 SV set. The x-axis shows the SV size, and the y-axis represents the number of SVs in different size ranges. We excluded translocations in the plots as the length of translocations are not defined by SV callers. INS: insertion, DEL: deletion, DUP: duplication, INV: inversion

The real SV datasets contain the SV calls of HG002 and HG005 from GIAB [35] **(**Table 1**)**. The HG002 sample includes 69× PacBio CLR, 28× CCS, and 47× ONT ultra-long reads data. The HG002 benchmark set has two tiers of SV calls. The Tier 1 benchmark set includes 12,745 sequence-resolved SVs [35]. The Tier 2 benchmark set contains 7001 imprecisely determined SVs [35]. Because the HG002 benchmark set only includes INSs and DELs, we generated an SV dataset with five types of SVs using the 30× PacBio CCS data from the HG005 sample and then evaluated the genotyping accuracy of the five methods (see “Methods” section for details). We observed a similar SV size distribution between the simulated and real SV sets (Fig. 1). Specifically, the frequency of SV decreases exponentially with increasing size for INSs, DELs, and DUPs (Fig. 1a-c and e-k). Moreover, we observed peaks at ~ 0.3 kb and 6 kb in the simulated (Fig. 1a, b), HG002 Tier1 (Fig. 1e, f), and HG005 (Fig. 1i, j) SV sets, reflecting the activities of Alu and LINE1 transposable elements in the human genome.

### Aligners and SV genotyping methods

The simulated and real LRS data were mapped to the human reference genome using NGMLR [37] and minimap2 [40]. We benchmarked five SV genotyping methods, including cuteSV [36], LRcaller [32], Sniffles [37], SVJedi [14], and VaPoR [38]. Table 2 lists the details of each SV genotyping method, including version, compatible aligners, applicable SV types, acceptable sequencing data types, input, and output. Among these five SV genotyping methods, cuteSV and Sniffles were originally developed as de novo SV callers, but can perform SV genotyping function using the -Ivcf option. LRcaller and SVJedi were developed as SV genotyper using LRS data. VaPoR is a long-read-based tool to visualize and genotype known SVs. cuteSV, LRcaller, and Sniffles are capable of genotyping all five types of SVs. SVJedi requires alternate allele sequences to genotype INSs and cannot genotype DUPs. VaPoR does not support genotyping TRAs. cuteSV, LRcaller, Sniffles, and VaPoR use BAM files from minimap2 or NGMLR as input. SVJedi takes sequencing data in the FASTA/FASTQ format or aligned PAF files from minimap2 as input (see the “Methods” section for details).

**Table 2:** Summary of SV genotyping methods

Category	cuteSV	LRcaller	Sniffles	SVJedi	VaPoR
Version	1.0.11	0.2	1.0.12a	1.1.0	NA
Aligner	minimap2, NGMLR	minimap2, NGMLR	minimap2, NGMLR	minimap2	minimap2, NGMLR
SV type	INSs, DELs, DUPs, INVs, TRAs	INSs, DELs, DUPs, INVs, TRAs	INSs, DELs, DUPs, INVs, TRAs	INSs, DELs, INVs, TRAs	INSs, DELs, DUPs, INVs
Data type	CLR, CCS, ONT	CLR, CCS, ONT	CLR, CCS, ONT	CLR, ONT	CLR, CCS, ONT
Input	REF, BAM, VCF	REF, BAM, VCF	BAM, VCF	REF, VCF, FASTA/FASTQ; PAF, VCF	REF, BAM, VCF/BED
Output	1 genotype	5 genotypes	1 genotype	1 genotype	1 genotype

REF: the human reference genome; BAM: alignment in BAM (binary alignment map) format; VCF: targeted SVs in VCF (variant call format); FASTA/FASTQ: sequencing data in FASTA or FASTQ format; PAF: alignment in PAF (pairwise mapping format); BED: targeted SVs in BED (browser extensible data) format. “NA” indicates the data are not available. LRcaller employs five different genotyping models: direct (AD), variant alignment (VA), joint (J), presence (PR), and reference aware variant alignment (VAr), resulting in five genotypes [32]. We used the genotypes of the default joint model when comparing it with other SV genotyping methods

### Evaluation of SV genotyping based on simulated data

**Fig. 2:**
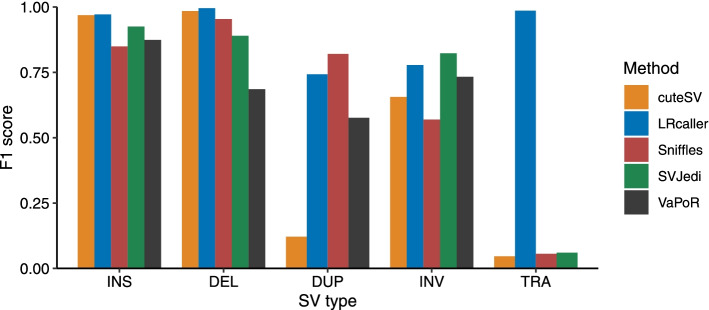
F1 scores of SV genotyping methods based on the simulated data. The x-axis is the SV type, and the y-axis shows the F1 score of each SV genotyping method. Performance was estimated on ~ 30× PacBio CLR data. The alignment files were generated by minimap2 because its output was compatible to all SV genotyping methods. SVJedi and VaPoR are inapplicable to DUPs and TRAs, respectively. INS: insertion, DEL: deletion, DUP: duplication, INV: inversion, TRA: translocation

First, we calculated the F1 scores of the five SV genotyping methods based on the simulated SV dataset that consisted of 15,453 SVs (7710 INSs, 7290 DELs, 167 DUPs, 72 INVs, and 214 TRAs). The benchmark results (Fig. 2, Fig. S1, and Table S1) showed that LRcaller achieved the highest F1 scores for INSs (0.97), DELs (0.99), and TRAs (0.99). Sniffles and SVJedi obtained the highest F1 scores for DUPs (0.82) and INVs (0.82) respectively.

### Evaluation of SV genotyping based on real data

**Fig. 3:**
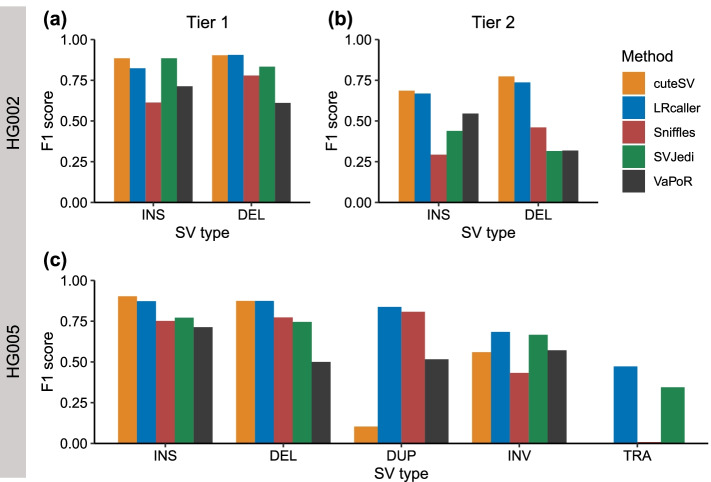
F1 scores of SV genotyping methods based on real data. **a** HG002 Tier 1 dataset. **b** HG002 Tier 2 dataset. **c** HG005 dataset. The x-axis represents the SV type, and the y-axis shows the F1 score of each SV genotyping method. Performance was estimated on ~ 30× PacBio CLR data. The alignment files were based on minimap2 because it was compatible to all SV genotyping methods. SVJedi and VaPoR are inapplicable to DUPs and TRAs, respectively. INS: insertion, DEL: deletion, DUP: duplication, INV: inversion, TRA: translocation

We next evaluated the performance of the five SV genotyping methods based on three real SV sets (the HG002 Tier 1, HG002 Tier 2, and HG005) (Fig. 3, Fig. S2, and Tables S2, S3 and S4). The results showed that for INSs and DELs in HG002 and HG005, cuteSV (INS: 0.69–0.90, DEL: 0.77–0.90) and LRcaller (INSs: 0.67–0.87, DELs: 0.74–0.91) had similar F1 scores across three real SV sets and outperformed other three genotyping methods (Fig. 3a–c). For DUPs, INVs, and TRAs in HG005 (Fig. 3c), LRcaller achieved higher F1 scores (DUPs: 0.84; INVs: 0.68; and TRAs: 0.47) than cuteSV (DUPs: 0.10, INVs: 0.56, and TRAs: 0.00), Sniffles (DUPs: 0.81, INVs: 0.43, and TRAs: 0.01), SVJedi (INVs: 0.67, TRAs: 0.34), and VaPoR (DUPs: 0.52, INVs: 0.57). Note that SVjedi cannot genotype DUP and VaPoR cannot genotype TRA.

### Evaluation of SV genotyping based on Mendelian Concordance Rate (MCR)

MCR provides an independent evaluation of accuracies of variant calling and genotyping based on trio data [41]. We evaluated the MCR of each SV genotyping method based on the SVs of an Ashkenazim trio (HG002, HG003, and HG004) and a Chinese trio (HG005, HG006, and HG007) **(**Table 3**)**. We used both HG002 Tier 1 and Tier 2 SV sets in the evaluation. Our analyses showed that LRcaller had the highest MCRs (0.98, 0.96, and 0.98), followed by SVJedi (0.97, 0.96, and 0.97), VaPoR (0.95, 0.92, and 0.96), Sniffles (0.91, 0.92, and 0.94), and cuteSV (0.92, 0.83, and 0.95) based on the SVs of the Tier 1 and Tier 2 of HG002, as well as HG005.

**Table 3:** Mendelian concordance of SV genotyping methods on trio datasets

SV genotyping method	MCR		
	HG002 Tier 1	HG002 Tier 2	HG005
cuteSV	0.92	0.83	0.95
LRcaller	0.98	0.96	0.98
Sniffles	0.91	0.92	0.94
SVJedi	0.97	0.96	0.97
VaPoR	0.95	0.92	0.96

The Ashkenazim trio includes son HG002, father HG003, and mother HG004. The Chinese trio includes son HG005, father HG006, and mother HG007. Performance was estimated based on ~ 30× PacBio CLR data. The alignment files were from minimap2 because its output was compatible with all SV genotyping methods

*MCR* Mendelian concordance rate

### Impact of SV size on SV genotyping

**Fig. 4:**
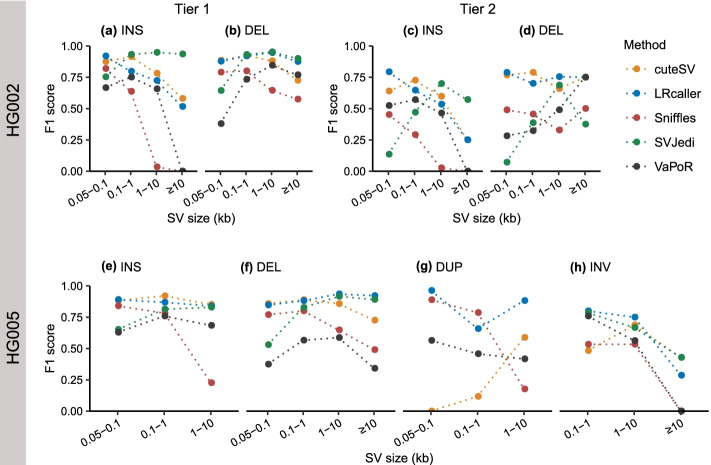
Impact of SV size on F1 scores of SV genotyping methods. **a, b** HG002 Tier 1 dataset. **c, d** HG002 Tier 2 dataset. **e–h** HG005 dataset. The x-axis represents SV size, and the y-axis shows the F1 score of each SV genotyping method in each size range. Performance was evaluated on 30× PacBio CLR data, and the alignment files were generated by minimap2. The TRA type has no definite size and is not included in the evaluation. INS: insertion, DEL: deletion, DUP: duplication, INV: inversion

We examined the impact of SV size on the F1 scores of SV genotyping methods based on three real SV sets. We observed that the F1 scores of different methods varied with SV size (Fig. 4). For example, with increasing of INS sizes from < 100 bp to ≥10 kb (Fig. 4a), the F1 scores of four methods decreased from 0.87 to 0.58 (cuteSV), 0.92 to 0.52 (LRcaller), 0.82 to 0.00 (Sniffles), and 0.67 to 0.00 (VaPoR). In contrast, we observed that the F1 scores of SVJedi increased from 0.75 to 0.93 when genotyping INSs from < 100 bp to ≥10 kb in size (Fig. 4a). For DELs (Fig. 4b, d, and f), the impacts of SV size on F1 scores were weaker compared to INSs (Fig. 4a, c, and e). In particular, we found an increase in F1 scores when genotyping DELs ≥100 bp in size compared to shorter ones, suggesting the DELs < 100 bp in size are more difficult to identify compared to longer ones (Fig. 4b, d, and f).

Furthermore, almost all genotyping methods achieved their best F1 scores for DUPs < 100 bp (except for cuteSV) (Fig. 4g). For example, with DUP sizes ranging from < 100 bp to ≥1 kb, the F1 scores of three methods decreased from 0.96 to 0.66 (LRcaller), 0.89 to 0.79 (Sniffles), and 0.56 to 0.46 (VaPoR). However, cuteSV showed a consistent increase in F1 scores with increasing DUP size, due to its poor performance when genotyping DUPs < 1 kb in size (F1 scores < 0.12). In addition, we observed that all genotyping methods achieved their lowest F1 scores when genotyping the INVs ≥ 10 kb (Fig. 4h).

### Impact of tandem repeat region on SV genotyping

**Fig. 5:**
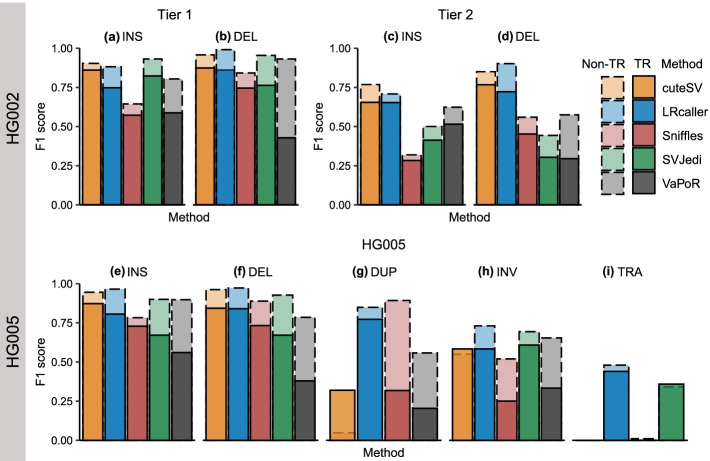
Stacked bar plots displaying impact of tandem repeat (TR) on the F1 scores of SV genotyping methods. **a, b** HG002 Tier 1 dataset. **c, d** HG002 Tier 2 dataset. **e–i** HG005 dataset. The x-axis represents SV genotyping methods, and the y-axis shows the F1 score of each SV genotyping method in different genomic regions. TR and non-TR mean SVs located in and outside of TR regions, respectively. Performance was evaluated on 30× PacBio CLR data and the alignment files were based on minimap2. INS: insertion, DEL: deletion, DUP: duplication, INV: inversion, TRA: translocation

We further assessed the F1 scores of five methods when genotyping the SVs located in tandem repeat (TR) regions based on the three real datasets. We observed that the F1 scores of each method for SVs located in TR regions were lower than SVs outside of TR regions (Fig. 5). For example, compared to INSs within TR regions (Fig. 5a), the F1 scores of five methods for INSs located within TR regions declined from 0.90 to 0.86 (cuteSV), 0.88 to 0.75 (LRcaller), 0.64 to 0.57 (Sniffles), 0.93 to 0.82 (SVJedi), and 0.80 to 0.59 (VaPoR). A similar trend was observed in the HG002 Tier 2 (Fig. 5c, d) and HG005 datasets (Fig. 5e–i). Moreover, we found that TR regions had less impact on genotyping INVs and TRAs (Fig. 5h, i) compared to other SV types.

### Impact of imprecise breakpoint on SV genotyping

**Fig. 6:**
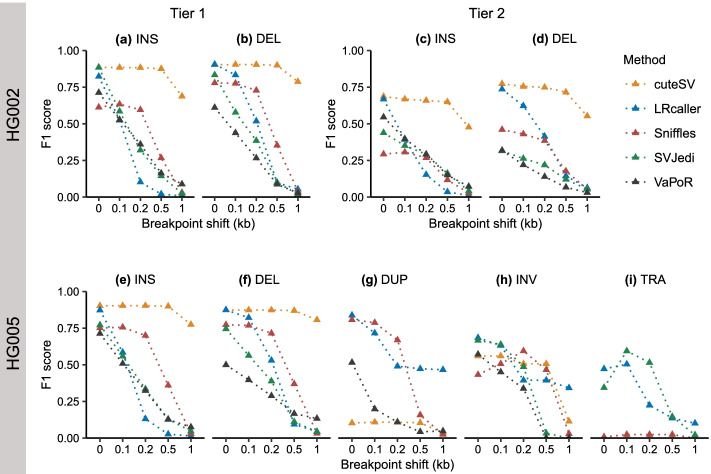
Impact of imprecise breakpoint on the F1 score of SV genotyping methods. **a, b** HG002 Tier 1 dataset. **c, d** HG002 Tier 2 dataset. **e–i** HG005 dataset. The x-axis is the offset (bp: base pair) between the original and shifted breakpoints, and the y-axis is the F1 score of each SV genotyping method in each breakpoint shift. Performance was evaluated on 30× PacBio CLR data and the alignment files were generated by minimap2. INS: insertion, DEL: deletion, DUP: duplication, INV: inversion, TRA: translocation

Previous studies have shown that SV detection methods often generate SVs with imprecise breakpoints [42] due to an enrichment of breakpoints in highly repetitive regions [4] and high sequencing error rates (5–15%) in LRS data [22]. To investigate the impact of imprecise breakpoints on the genotyping accuracy of each method, we shifted the breakpoints of SVs in three real SV sets by 100 bp, 200 bp, 500 bp, and 1000 bp. Overall, we observed that the F1 scores of all methods decrease with increasing breakpoint shift (Fig. 6). For example, for INSs with breakpoint shifts ranging from 100 to 1000 bp (Fig. 6a), the F1 scores of five methods declined from 0.88 to 0.69 (cuteSV), 0.52 to 0.01 (LRcaller), 0.63 to 0.03 (Sniffles), 0.59 to 0.03 (SVJedi), and 0.53 to 0.09 (VaPoR).

Moreover, the robustness of genotyping methods varies when handling different breakpoint shifts. For example, for INSs and DELs in HG002 and HG005 (Fig. 6a–f), cuteSV was less sensitive to breakpoint shift than other methods, which showed a decrease in F1 scores < 0.04 when breakpoint shifts ≤500 bp. For DUPs, INVs, and TRAs in HG005 (Fig. 6g–i), we found that LRcaller was quite robust when genotyping SVs with different breakpoint shifts.

### Impacts of aligner and sequencing data on SV genotyping

Next, we compared the F1 scores of SV genotyping methods under different combinations of aligners (minimap2 and NGMLR) and sequencing data (PacBio CLR, PacBio CCS, and ONT). We found that a combination of minimap2 and PacBio CCS data outperformed other combinations (Table 4). For example, the F1 scores of cuteSV, LRcaller, SVJedi, and VaPoR based on the combination of minimap2 and PacBio CCS data were 0.03–0.06 higher than other combinations when genotyping HG002 Tier 1 SVs. In particular, Sniffles showed a 0.18 (from 0.65 to 0.83) increase in F1 score based on the combination of minimap2 and PacBio CCS data compared to other combinations. For the HG002 Tier 2 and HG005 datasets (Tables S5, S6), the F1 scores of genotyping methods showed a similar pattern to the HG002 Tier 1 dataset.

**Table 4:** F1 scores of SV genotyping methods based on different aligners and sequencing data

SV genotyping method	Aligner	Sequencing data			Max-Min
		CLR	ONT	CCS	
cuteSV	minimap2	0.89	**0.93**	**0.93**	0.06
	NGMLR	0.87	0.91	0.91	
LRcaller	minimap2	0.86	0.85	**0.88**	0.03
	NGMLR	0.87	0.87	0.87	
Sniffles	minimap2	0.68	0.81	**0.83**	0.18
	NGMLR	0.65	0.73	0.76	
SVJedi	minimap2	**0.86**	0.81	NA	0.05
VaPoR	minimap2	0.67	**0.71**	**0.71**	0.04
	NGMLR	0.67	**0.71**	**0.71**	

Impact of aligner (minimap2 and NGMLR) and sequencing data (PacBio CLR, PacBio CCS, and ONT) on the F1 score of each genotyping method based on the HG002 Tier 1 dataset. Performance was evaluated on 30× HG002 sequencing data. SVJedi does not support the output from NGMLR. “NA” indicates the data is not available. The bold black number is the highest F1 score for each SV genotyping method. The “Max-Min” column represents the maximum F1 score minus the minimum F1 score for each SV genotyping method under different combinations of aligners and sequencing data

### Impact of depth of coverage on SV genotyping

**Fig. 7:**
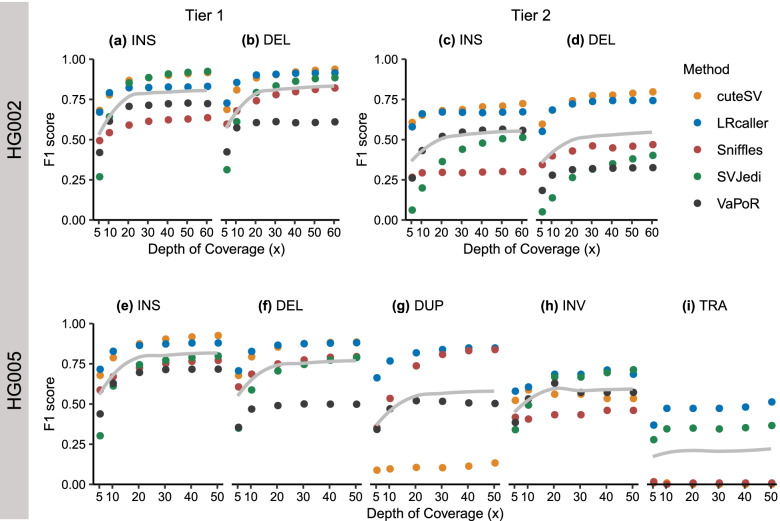
Impact of depth of coverage on the F1 score of SV genotyping methods. **a, b** HG002 Tier 1 dataset. **c, d** HG002 Tier 2 dataset. **e–i** HG005 dataset. The x-axis represents depth of coverage, and the y-axis indicates the F1 score of each SV genotyping method in different sequencing depths. The gray line in each sub-figure represents the smooth curve generated by locally weighted regression using the loess function in R. For each dataset, we downsampled the sequencing data of HG002 and HG005 to different depths using SAMtools [43]. SVJedi and VaPoR cannot genotype DUP and TRA, respectively. INS: insertion, DEL: deletion, DUP: duplication, INV: inversion, TRA: translocation

To explore the impact of depth of coverage on SV genotyping, we downsampled ~ 69× HG002 PacBio CLR data to 60×, 50×, 40×, 30×, 20×, 10×, and 5× coverages and ~ 57× HG005 PacBio CLR data to 50×, 40×, 30×, 20×, 10×, and 5× coverages, respectively. Then, we aligned the downsampled data to the human reference genomes (hs37d5 and GRCh38) using minimap2 and calculated the F1 scores of each method at different depth of coverages. The results showed that the F1 scores of all genotyping methods in the present study rapidly increased at 5–20× with increasing coverages (Fig. 7). For example, when depth of coverage increased from 5× to 20× (Fig. 7a), the F1 scores of all methods for INSs increased by 0.10–0.58 (i.e., cuteSV: 0.68 to 0.87, LRcaller: 0.67 to 0.82, Sniffles: 0.49 to 0.59, SVJedi: 0.27 to 0.85, and VaPoR: 0.42 to 0.71). However, when depth of coverage was increased from 20× to 60×, the F1 scores of INSs only increased by 0.01–0.05 (i.e., cuteSV: 0.87 to 0.92, LRcaller: 0.82 to 0.83, Sniffles: 0.59 to 0.64, SVJedi: 0.85 to 0.92, and VaPoR: 0.71 to 0.72). We observed such a pattern in genotyping other types of SVs (Fig. 7b–i). Our results that a slight increment in performance after depth of coverage > 20× were also found in a prior study of SV calling methods using nanopore sequencing data [44].

### Evaluation of computational resource consumption

**Fig. 8:**
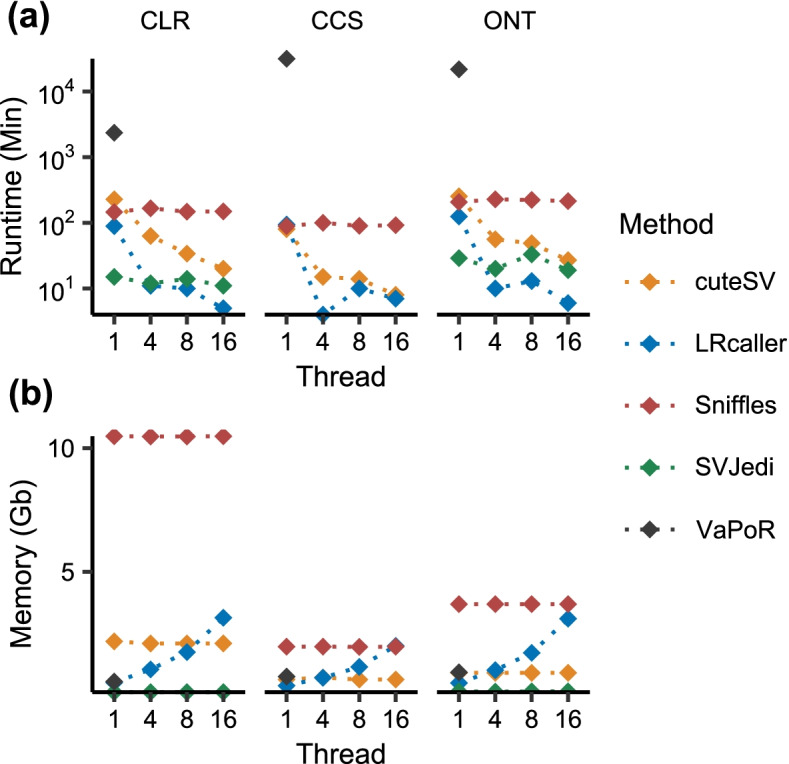
Computational resource consumption of SV genotyping methods. **a** Runtimes (Min: minutes). **b** Memory cost (Gb: gigabyte). cuteSV, LRcaller, Sniffles, and SVJedi were run using at 1, 4, 8, and 16 CPU threads. VaPoR does not support multiple thread mode

We finally compared computational resource consumption for each SV genotyping method based on the HG002 Tier 1 dataset using 30× PacBio CLR, PacBio CCS, and ONT sequencing data (Fig. 8). We found that SVJedi showed the shortest running time under single thread mode and requires the lowest memory no matter in single or multiple thread modes (Fig. 8a, b). In addition, LRcaller is the most efficient with regard to running time under multiple thread mode compared to other methods (Fig. 8a).

## Discussion

LRS, with average read lengths over 10 kb, has significantly boosted the study of SVs in humans and has been widely used in basic science [30, 32, 45] and clinical studies [31, 33, 46]. In this study, we comprehensively benchmarked five state-of-the-art LRS-based SV genotyping methods (including cuteSV, LRcaller, Sniffles, SVJedi, and VaPoR) using both the simulated and real LRS datasets.

We observed that LRS-based genotyping methods not only genotyped higher numbers of SVs, but also yielded better accuracy than the SRS-based methods. For example, cuteSV and LRcaller genotyped 99.98% (12,743 of 12,745) and 100% (12,745 of 12,745) of Tier 1 SVs of HG002. In comparison, a prior study [35] showed that vg and paragraph, two SRS-based methods, genotyped 88.76% (11,313 of 12,745) and 93.17% (11,874 of 12,745). In addition, we found 10,074 consistent genotypes for cuteSV, LRcaller, and Tier 1 SVs of HG002, which is higher than the number (6612) between vg [23] and paragraph [22], suggesting a better congruence of LRS-based genotyping methods than the SRS-based methods. Second, our analysis revealed that LRS-based genotyping methods are quite robust when genotyping SVs with imprecise breakpoints (Fig. 6). For example, cuteSV showed a < 0.1 decrease of F1 scores when genotyping INSs and DELs with breakpoint shifts ≤500 bp in size. In contrast, previous studies have shown that SRS-based genotyping methods can only tolerate breakpoint shifts of up to 10 bp in size [22, 23]. Third, LRS-based genotyping methods are capable of genotyping all types of SVs, particularly INSs or TRAs, which are difficult to genotype using SRS-based SV genotyping methods [15] (Figs. 2 and 3). In addition, our down-sampling experiments reveal high performance of genotyping methods from 20× coverage. For example, cuteSV with 20× coverage obtained a F1-score of 0.87, while tripling the coverage only resulted in a 0.05 increase of F1-score when genotyping INS. We observe a similar pattern when genotyping other types of SV. Finally, we found the LRS-based genotyping methods performed best using PacBio CCS data (Table 4), which could result from an improved sequence alignment at highly repetitive or segmental duplication regions with the help of base-calling accuracy of HiFi reads [47].

We also observed that a strong impact of SV type on the performance of LRS-based SV genotyping methods. Better genotyping accuracies were observed for INSs and DELs than other types of SVs. In particular, we found that all LRS-based genotyping methods had the lowest accuracy (F1 scores ≤0.47) when genotyping TRAs (Fig. 3c). The observation may be relevant to the fact that TRAs involve fragments of two chromosomal segments that are often accompanied by some additional rearrangements of up to millions of base pairs such as deletions and duplications [48], which are difficult to use in read alignment and in SV calling and genotyping. In addition, we cannot exclude that some of TRAs in the HG005 SV dataset are false positive. Compared to sequenced-resolved SVs (Fig. 3a), all genotyping methods had lower F1 scores when genotyping the imprecisely determined SVs (Fig. 3b). This may be because these regions with clustered SVs are difficult to sequence and align reads correctly [22] or SV genotyping methods cannot properly distinguish the supporting reads for each SV when they are too close to each other [23]. Our analyses also identified potential method-specific limitations. For example, cuteSV had poor performance for DUPs ≤100 bp in size (F1 score: 0.10) (Fig. 4g).

## Limitations and conclusions

Here, we comprehensively assessed the performance of five SV genotyping methods using the simulated and real LRS datasets. The four datasets we employed have specific limitations in assessing SV genotyping. The HG002 dataset contains only INSs and DELs. The performance of the genotyping methods on other types of SVs were evaluated using simulated and HG005 SV datasets. However, the simulated dataset cannot fully reflect the complexity of real human genomes. In addition, the synthetic reads were normally generated based on simple generative models. Although we only included the SVs that were supported by at least 2 callers when generating the HG005 benchmark SVs, this dataset is not well-curated and is likely to be biased to the SVs from easy-to-detect genomic regions. Further, the low numbers of DUPs (296), INVs (38), and TRAs (125) in the HG005 benchmark dataset may hinder our ability to comprehensively evaluate the performance of genotyping methods on these SV types. Nevertheless, we highlight the challenges or limitations of the current LRS-based SV genotyping methods. These benchmark results will facilitate the application and improvement of SV genotyping methods based on long reads.

## Methods

### Simulation dataset generation

VISOR v1.1 [39] was used for SV simulation. We extracted 7710 INSs, 7290 DELs, 167 DUPs, and 72 INVs from NA19240 sample callsets [49] (nstd152 in dbVAR [50]), and 214 TRAs from KWB1 sample callsets [51] (nstd107 in dbVAR). The downloaded SVs were integrated into the human reference genome (GRCh38) to build one in silico donor genome, which was then used as input of VISOR (HACk mode) for SV simulation. We also simulated 30x Pacbio CLR sequencing data using VISOR LASeR mode with parameters --read_type pacbio --error_model pacbio2016 --qscore_model pacbio2016 as well as other default parameters. Note that we used odd- and even-numbered autosomes for the generation of homozygous and heterozygous SVs, respectively. This was implemented using different purity values in VISOR LASeR mode.

The command lines for data simulation using VISOR are in Supplementary Materials.

### GIAB dataset

The GIAB HG002 benchmark set was downloaded from NCBI (https://ftp-trace.ncbi.nlm.nih.gov/giab/ftp/data/AshkenazimTrio/analysis/NIST_SVs_Integration_v0.6/HG002_SVs_Tier1_v0.6.vcf.gz). The SVs in the benchmark set were classified into two categories, Tier 1 and Tier 2. The Tier 1 benchmark set contained 7281 and 5464 isolated and sequence-resolved INSs and DELs respectively. The Tier 2 benchmark set consists of 7001 clustered SVs with determined genotypes. The sequencing data for HG002 (PacBio CLR, PacBio CCS, and ONT data), HG003 (PacBio CLR), and HG004 (PacBio CLR) samples were downloaded from GIAB FTP server (ftp://ftp.ncbi.nlm.nih.gov/giab/ftp/data/AshkenazimTrio/).

We downloaded the HG005 PacBio CCS read data from NCBI BioProject database (accession number PRJNA540706) and converted the SRA files to FASTQ format using fastq-dump of SRA Toolkit (http://www.ncbi.nlm.nih.gov/Traces/sra/sra.cgi?view=toolkit_doc&f=fastq-dump). The sequencing data of HG005 (PacBio CLR), HG006 (PacBio CLR), and HG007 (PacBio CLR) were downloaded from GIAB FTP site (ftp://ftp.ncbi.nlm.nih.gov/giab/ftp/data/ChineseTrio/). The CCS reads were mapped to human reference genome (GRCh38) using PBMM2 v1.4.0 (https://github.com/PacificBiosciences/pbmm2) with CCS mode (−-preset CCS) and minimap2 (−ax asm20 --MD -Y) respectively. We conducted SV calling using three SV callers, including PBSV v2.4.0 (https://github.com/PacificBiosciences/pbsv), SKSV v1.0.3 [52], and DeBreak v1.0.2 [53]. The PBSV discover stage used the BAM file from PBMM2 and was run using --tandem-repeats parameter (https://github.com/PacificBiosciences/pbsv/blob/master/annotations/human_GRCh38_no_alt_analysis_set.trf.bed). The PBSV call stage was run with parameters “--ccs -A 3 -O 3 -P 20 --gt-min-reads 3 -t INS, DEL, DUP, INV, BND”. For SKSV, it used sequencing files in FASTQ format as input. The index and alignment stages were performed with default parameters. SKSV call stage was run with parameter “--genotype” to generate genotypes. DeBreak used the BAM files from minimap2 as input and was run with full function mode. The BND calls from PBSV and SKSV were considered as TRA. We used the SVs that are > 50 bp, with the FILTER “PASS” tag, and determined genotype in the further analyses. Then, the SVs of three callers were merged using Jasmine v.1.0.1 [54] with parameters “--ignore_strand --output_genotypes”. “ignore_strand” allows to merge SVs on different strands since that the “STRANDS” tag is frequently missing in VCF files of SV callers. “--output_genotypes” outputs the genotypes of the consensus SV in the VCF files from different callers. Finally, we kept SVs with at least two consistent genotypes in the merged VCF file for evaluation. The benchmark SV dataset of HG005 contains 8867, 8121, 296, 38, and 125 INS, DELs, DUPs, INVs, and TRAs.

### Read mapping and SV genotyping

The simulated PacBio CLR data were mapped to the human reference genome GRCh38 (only autosomal and sex chromosomes were included) using minimap2 v2.17-r941. PacBio CLR data of HG005 trio (HG005, HG006, and HG007) and PacBio CCS data of HG005 were also mapped to the human reference genome GRCh38 using using minimap2 v2.17-r941 and NGMLR v0.2.7. The PacBio CLR (HG002, HG003, and HG004), PacBio CCS (HG002) and ONT (HG002) datasets were mapped to the human reference genome (hs37d5) using minimap2 v2.17-r941 and NGMLR v0.2.7, respectively. We used the parameters “-ax map-pb --MD -Y”, “-ax asm20 --MD -Y”, and “-a -z 600,200 -x map-ont --MD -Y” to mapped PacBio CLR data, PacBio CCS data and ONT data respectively in minimap2. We used the parameter “-x pacbio” to align PacBio CLR and PacBio CCS data and “-x ont” to align ONT data in NGMLR. SAMtools was employed for read extraction, sorting, indexing, and downsampling of BAM files.

The specific parameters of each SV genotyping method are described below:

For cuteSV v1.0.11, we used the parameters “--max_cluster_bias_INS 100 --diff_ratio_merging_INS 0.3 --max_cluster_bias_DEL 200 --diff_ratio_merging_DEL 0.5 -mi 500 -md 500 -s 3 --genotype -Ivcf -L 150000” was run on PacBio CLR data, the parameters “--max_cluster_bias_INS 100 --diff_ratio_merging_INS 0.3 --max_cluster_bias_DEL 100 --diff_ratio_merging_DEL 0.3 -mi 500 -md 500 -s 3 --genotype -Ivcf -L 150000” was run on ONT data, and the parameters “--max_cluster_bias_INS 1000 --diff_ratio_merging_INS 0.9 --max_cluster_bias_DEL 1000 --diff_ratio_merging_DEL 0.8 -mi 500 -md 500 -s 3 --genotype -Ivcf -L 150000” was run on PacBio CCS data.

For LRcaller v0.2, default parameters were used for PacBio CLR, PacBio CCS, and ONT datasets. LRcaller used five genotyping models and provided five genotypes. The models were in order as follows: direct (AD), variant alignment (VA), joint (J), presence (PR), and reference aware variant alignment (VAr). The results of default J model were chosen to compare with other SV genotyping methods.

For Sniffles v1.0.12a, the parameter “--Ivcf” was employed in genotyping using the mapping results of PacBio CLR and ONT. We used the parameters “--Ivcf --skip_parameter_estimation” for PacBio CCS datasets. When running SV genotyping, Sniffles converted DUP to INS and TRA to INV, respectively. Thus, before genotyping, we separated the benchmark SVs into different VCF files based on SV type. After genotyping, the corresponding SV type was converted back.

For SVJedi v1.1.0, the configuration -d “pb” and “ont” was applied to PacBio CLR and ONT datasets, respectively. SVJedi v1.1.0 allowed long-read sequencing data in FASTQ/FASTA format or aligned reads in PAF format as input.

For VaPoR, the mode “vapor bed” was used for genotyping based on PacBio CLR, PacBio CCS, and ONT data. The output files were converted to VCF format using an in-house shell script.

### Evaluation factors for SV genotyping methods

#### The evaluation based on F1 score

Truvari v2.0.0-dev (https://github.com/ACEnglish/truvari) with “truvari bench --gtcomp (genotype comparison)” mode was used to calculate the precision, recall, and F1 score of the callsets generated by SV genotyping methods. Precision is defined as the number of correct genotype calls divided by all determined genotypes (0/0, 0/1, and 1/1) for each genotyping method. Recall is defined as the number of correct genotype calls divided by the number of benchmark SVs for each genotyping method. A F1 score was calculated using the following equation:

$$\mathrm{F}1\ \mathrm{score}=\frac{2\ast \mathrm{precision}\ast \mathrm{recall}}{\mathrm{precision}+\mathrm{recall}}$$

#### The evaluation of Mendelian concordance

We used BCFtools v1.14 (https://samtools.github.io/bcftools/) plugin “mendelian” with default parameters to count Mendelian concordance for each trio dataset. The HG002 Tier 1 and Tier 2 SV sets on autosomes were genotyped using ~ 30× PacBio CLR data from the Ashkenazim Trio, including son (HG002), father (HG003), and mother (HG004). The HG005 SV set was genotyped using ~ 30× PacBio CLR data from the Chinese Trio, including son (HG005), father (HG006), and mother (HG007). We calculated the proportion of SVs following Mendelian concordance genotypes in all estimated genotypes to evaluate Mendelian concordance rate (MCR).

$$\mathrm{MCR}=\frac{\mathrm{Mendelian}\ \mathrm{concordance}\ \mathrm{genotypes}}{\mathrm{Estimated}\ \mathrm{genotypes}\ }$$

### Evaluation of breakpoints located in tandem repeat regions

We downloaded the tandem repeat tracks of hg37d5 (https://github.com/PacificBiosciences/pbsv/blob/master/annotations/human_hs37d5.trf.bed) and of GRCh38 (https://github.com/PacificBiosciences/pbsv/blob/master/annotations/human_GRCh38_no_alt_analysis_set.trf.bed). We identified the SVs that locate in the TR regions of the reference genome using the intersect mode in BEDTools v2.30.0 [55].

### Evaluation of breakpoint shifting

We shifted the breakpoints of SV calls of HG002 (Tier 1 and Tier 2) and HG005 SV calls using a customized script. We randomly shifted the breakpoints of SVs 100 bp, 200 bp, 500 bp, and 1000 bp up- or down-stream. The modified SV sets were genotyped based on 30× PacBio CLR data.

### Running time and memory consumption

The command “/usr/bin/time -v” of the Linux operating system was employed to record runtime and memory consumptions at the SV genotyping step. We extracted the elapsed (wall clock) time and the maximum resident set size from the output files and used these as the elapsed runtime and memory consumption, respectively.

## Supplementary Information

**Additional file 1: Fig. S1.** Precision and recall on the simulated dataset. **Fig. S2.** Precision and recall on the real datasets. **Table S1.** Genotype contingency table on the simulated dataset. **Table S2.** Genotype contingency table on the HG002 Tier 1 dataset. **Table S3.** Genotype contingency table on the HG002 Tier 2 dataset. **Table S4.** Genotype contingency table on the HG005 dataset. **Table S5.** Impacts of aligner and sequencing data on genotyping based on HG002 Tier 2 dataset. **Table S6.** Impacts of aligner and sequencing data on genotyping based on HG005 dataset. **Supplementary Notes**

## Data Availability

All VCF files generated by SV genotyping methods can be viewed and downloaded in The National Omics Data Encyclopedia (NODE) website using the following link: https://www.biosino.org/node/analysis/detail/OEZ008314.
