# Supplementary material for: Comprehensive evaluation of structural variant genotyping methods based on long-read sequencing data
Source: BMC Genomics. 2022 Apr 23;23:324. doi: 10.1186/s12864-022-08548-y (PMC9034514; doi:10.1186/s12864-022-08548-y)
Supplement: Supplementary file 1 — Additional file 1: Fig. S1. Precision and recall on the simulated dataset. Fig. S2. Precision and recall on the real datasets. Table S1. Genotype contingency table on the simulated dataset. Table S2. Genotype contingency table on the HG002 Tier 1 dataset. Table S3. Genotype contingency table on the HG002 Tier 2 dataset. Table S4. Genotype contingency table on the HG005 dataset. Table S5. Impacts of aligner and sequencing data on genotyping based on HG002 Tier 2 dataset. Table S6. Impacts of aligner and sequencing data on genotyping based on HG005 dataset. Supplementary Notes [file 12864_2022_8548_MOESM1_ESM.docx]

Supplementary Material for

Comprehensive evaluation of structural variant genotyping methods based on long-read sequencing data

**Content**

[Fig. S1. Precision and recall on the simulated dataset 2](#_Toc99551190)

[Fig. S2. Precision and recall on the real datasets 3](#_Toc99551191)

[Table S1. Genotype contingency table on the simulated dataset. 4](#_Toc99551192)

[Table S2. Genotype contingency table on the HG002 Tier 1 dataset 4](#_Toc99551193)

[Table S3. Genotype contingency table on the HG002 Tier 2 dataset 4](#_Toc99551194)

[Table S4. Genotype contingency table on the HG005 dataset 4](#_Toc99551195)

[Table S5. Impacts of aligner and sequencing data on genotyping based on HG002 Tier 2 dataset 5](#_Toc99551196)

[Table S6. Impacts of aligner and sequencing data on genotyping based on HG005 dataset 6](#_Toc99551197)

[Supplementary Notes 7](#_Toc99551198)


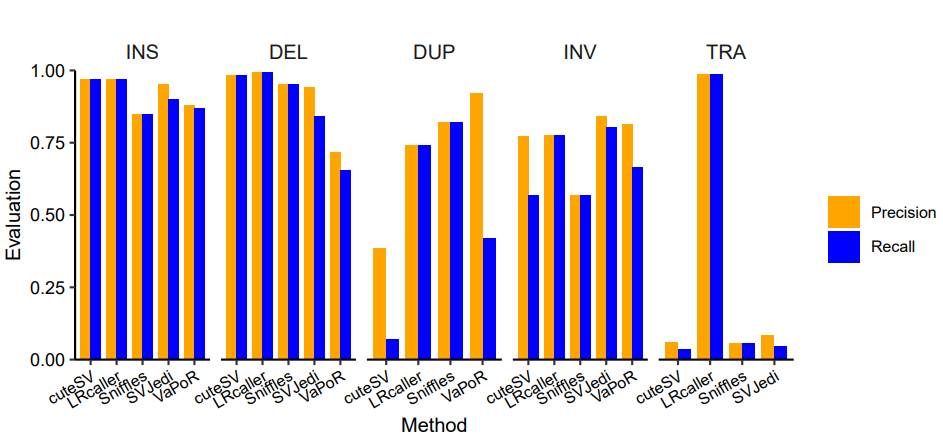


Fig. S1. Precision and recall rate of different methods based on the simulated dataset**.** The x-and y-axis shows SV genotyping methods and the precision or recall rates of each method. We simulated ~30× PacBio CLR data using VISOR. The alignment files were generated by minimap2 SVJedi and VaPoR can not genotype DUPs and TRAs respectively. INS: insertion, DEL: deletion, DUP: duplication, INV: inversion, TRA: translocation.


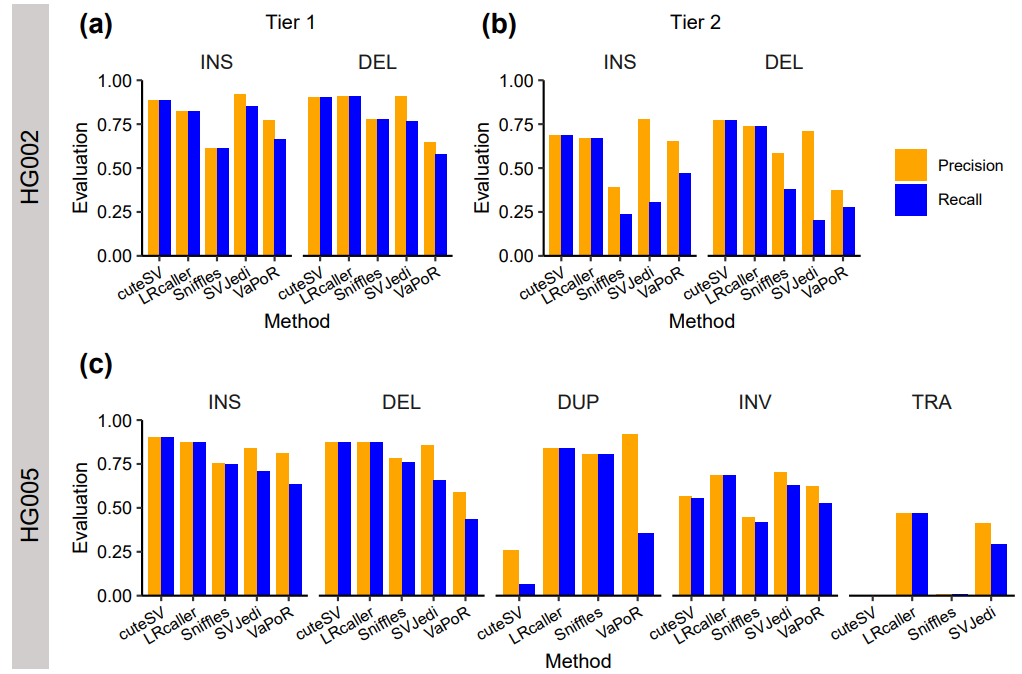


Fig. S2. Precision and recall rates of different methods based on the real datasets**.** (a) The HG002 Tier 1 dataset; (b) the HG002 Tier 2 dataset; (c) the HG005 dataset. The x-axis indicates SV genotyping methods, and the y-axis shows the precision or recall rates of each method. Performance was estimated on ~30× PacBio CLR data. The alignment files were generated by minimap2. SVJedi and VaPoR cannot genotype DUPs and TRAs, respectively. INS: insertion, DEL: deletion, DUP: duplication, INV: inversion, TRA: translocation.

Table S1. Genotype contingency table on the simulated dataset. The simulated genotypes were used as ground truth. The numbers highlighted in grey are consistent genotypes between the simulated SV set and SV genotyping methods. The numbers in the “./.” column are the count of genotypes that each genotyping method fails to determine. “NA” indicates the data is not available.

Table S2. Genotype contingency table on the HG002 Tier 1 dataset**.** We used the genotypes from the Tier 1 SV set of HG002 as the ground truth. We highlighted the numbers of consistent genotypes between the Tier 1 SV set and SV genotyping methods in grey. The “./.” column shows the numbers of genotype that each method fails to determine.

Table S3. Genotype contingency table on the HG002 Tier 2 dataset**.** We used the genotypes from the Tier 2 SV set of HG002 as the ground truth. We highlighted the numbers of consistent genotypes between the Tier 2 SV set and SV genotyping methods in grey. The “./.” column shows the numbers of genotype that each method fails to determine.

Table S4. Genotype contingency table on the HG005 dataset**.** We used the genotypes from the HG005 SV set as the ground truth. We highlighted the numbers of consistent genotypes between the HG005 SV set and each genotyping method in grey. The “./.” column shows the numbers of genotype that each method fails to determine. “NA” indicates the data is not available.

Table S5. Impacts of aligner and sequencing data on genotyping based on HG002 Tier 2 dataset**.** Performance was evaluated on 30× HG002 LRS data (PacBio CLR, PacBio CCS, and ONT data) with two alignments (minimap2 and NGMLR). SVJedi does not support the output of NGMLR. “NA” indicates the data is not available. The bold black numbers are the highest F1 score for each genotyping method. The “Max-Min” column is the maximum F1 score minus the minimum F1 score for each SV genotyping method under different combinations of aligners and sequencing data.

| SV genotyping method | Aligner | Sequencing data | | | Max-Min |
| --- | --- | --- | --- | --- | --- |
|  |  | CLR | ONT | CCS |  |
| cuteSV | minimap2 | 0.72 | 0.73 | **0.82** | 0.11 |
|  | NGMLR | 0.71 | 0.73 | 0.79 |  |
| LRcaller | minimap2 | 0.70 | 0.71 | **0.72** | 0.02 |
|  | NGMLR | 0.71 | 0.72 | 0.70 |  |
| Sniffles | minimap2 | 0.36 | 0.44 | **0.49** | 0.17 |
|  | NGMLR | 0.32 | 0.35 | 0.40 |  |
| SVJedi | minimap2 | **0.39** | **0.39** | NA | 0.00 |
| VaPoR | minimap2 | 0.45 | **0.53** | 0.51 | 0.08 |
|  | NGMLR | 0.45 | **0.53** | 0.51 |  |

Table S6. Impacts of aligner and sequencing data on genotyping based on HG005 dataset**.** Performance was evaluated on 30× HG005 LRS data (PacBio CLR and CCS data) with two alignments (minimap2 and NGMLR). SVJedi is unable to run with NGMLR. The ONT data of HG005 is not available. The bold numbers are the highest F1 score for each genotyping method. The “Max-Min” column means the maximum F1 score minus the minimum F1 score for each SV genotyping method under different combinations of aligners and sequencing data.

| SV genotyping method | Aligner | Sequencing data | | | Max-Min |
| --- | --- | --- | --- | --- | --- |
|  |  | CLR | ONT | CCS |  |
| cuteSV | minimap2 | 0.87 | NA | **0.92** | 0.07 |
|  | NGMLR | 0.85 | NA | 0.88 |  |
| LRcaller | minimap2 | 0.87 | NA | **0.91** | 0.06 |
|  | NGMLR | 0.85 | NA | 0.88 |  |
| Sniffles | minimap2 | 0.76 | NA | **0.81** | 0.11 |
|  | NGMLR | 0.70 | NA | 0.76 |  |
| SVJedi | minimap2 | 0.61 | NA | NA | NA |
| VaPoR | minimap2 | 0.61 | NA | **0.70** | 0.10 |
|  | NGMLR | 0.60 | NA | **0.70** |  |

Supplementary Notes

1. **Benchmark dataset**

**1.1 Simulated dataset**

VISOR HACk -g {reference.fa} -b {sv_input.bed} -o {hack_dir}

VISOR LASeR -g {reference.fa} -s {hack_dir} -b {purity.bed} -o {laser_dir} --read_type pacbio --error_model pacbio2016 --qscore_model pacbio2016

**1.2 HG002 dataset**

wget <ftp://ftp-trace.ncbi.nlm.nih.gov/giab/ftp/data/AshkenazimTrio/analysis/NIST_SVs_Integration_v0.6/HG002_SVs_Tier1_v0.6.vcf.gz>

**1.3 HG005 dataset**

PBSV:

pbmm2 align --sort --preset CCS --sample {sample} {reference.fa} {sample.fq} {sample.bam}

pbsv discover --tandem-repeats {trf.bed} {sample.bam} {sample.svsig.gz}

pbsv call --ccs -A 3 -O 3 -P 20 --gt-min-reads 3 -t INS,DEL,DUP,INV,BND {reference.fa} {sample.svsig.gz} {sample.vcf}

SKSV:

SKSV index {reference.fa} {index_route}

SKSV aln {index_route} {sample.fq}

SKSV call {aln.svseg} {reference.fa} {sample.vcf} {work_dir} --genotype

DeBreak:

minimap2 -ax asm20 {reference.fa} {sample.fq} --MD -Y -R '@RG\tID:{sample}' -o {sample.sam}

samtools view -bS {sample.sam} | samtools sort -O BAM - > {sample.bam}

debreak --bam {sample.bam} -o {debreak_dir} --rescue_large_ins --rescue_dup --poa --ref {reference.fa}

1. **Read alignment**

**2.1 minimap2**

Pacbio CLR:

minimap2 -ax map-pb {reference.fa} {sample.fq} --MD -Y -R '@RG\tID:{sample}' -o {sample.sam}

Pacbio CCS:

minimap2 -ax asm20 {reference.fa} {sample.fq} --MD -Y -R '@RG\tID:{sample}' -o {sample.sam}

ONT:

minimap2 -ax map-ont {reference.fa} {sample.fq} -z 600,200 --MD -Y -R '@RG\tID:{sample}' -o {sample.sam}

**2.2 ngmlr**

Pacbio CLR/CCS:

ngmlr -r {reference.fa} -q {sample.fq} -x pacbio -o {sample.sam}

ONT:

ngmlr -r {reference.fa} -q {sample.fq} -x ont -o {sample.sam}

1. **BAM sorting and down-sampling**

samtools view -bS {sample.sam} | samtools sort -O BAM - > {sample.bam} && samtools index {sample.bam}

samtools view -bS -s {ratio} {sample.bam} > {sample_ratio.bam} && samtools index {sample_ratio.bam}

1. **SV genotyping**

**4.1 cuteSV:**

Pacbio CLR:

cuteSV --max_cluster_bias_INS 100 --diff_ratio_merging_INS 0.3 --max_cluster_bias_DEL 200 --diff_ratio_merging_DEL 0.5 -mi 500 -md 500 -s 3 --genotype -Ivcf {sample.vcf} -S {sample} -L 150000 {sample.bam} {reference.fa} {sample_gt.vcf} {work_dir}

Pacbio CCS:

cuteSV --max_cluster_bias_INS 1000 --diff_ratio_merging_INS 0.9 --max_cluster_bias_DEL 1000 --diff_ratio_merging_DEL 0.8 -mi 500 -md 500 -s 3 --genotype -Ivcf {sample.vcf} -S {sample} -L 150000 {sample.bam} {reference.fa} {sample_gt.vcf} {work_dir}

ONT:

cuteSV --max_cluster_bias_INS 100 --diff_ratio_merging_INS 0.3 --max_cluster_bias_DEL 100 --diff_ratio_merging_DEL 0.3 -mi 500 -md 500 -s 3 --genotype -Ivcf {sample.vcf} -S {sample} -L 150000 {sample.bam} {reference.fa} {sample_gt.vcf} {work_dir}

**4.2 LRcaller:**

Pacbio CLR/Pacbio CCS/ONT:

LRcaller -fa {reference.fa} -a seqan {sample.bam} {sample.vcf} {sample_gt.vcf}

**4.3 Sniffles**

Pacbio CLR/ONT:

sniffles -m {sample.bam} -v {sample_gt.vcf} --Ivcf {sample.vcf}

Pacbio CCS:

sniffles --skip_parameter_estimation -m {sample.bam} -v {sample_gt.vcf} --Ivcf {sample.vcf}

**4.4 SVJedi**

Pacbio CLR:

python3 svjedi.py -d pb -v {sample.vcf} -r {reference.fa} -i {sample.fq} -o {sample_gt.vcf}

ONT:

python3 svjedi.py -d ont -v {sample.vcf} -r {reference.fa} -i {sample.fq} -o {sample_gt.vcf}

**4.5 VaPoR**

Pacbio CLR/ Pacbio CCS/ONT:

vapor bed --sv-input {input.bed} --output-path {vapor_dir} --output-file {output.file} --reference {reference.fa} --pacbio-input {sample.bam}

1. **Benchmarking**

bgzip {sample_gt.vcf} > {sample_gt.vcf.gz} && tabix {sample_gt.vcf.gz}

bgzip {bench_gt.vcf} > {bench_gt.vcf.gz} && tabix {bench_gt.vcf.gz}

truvari bench -b {bench_gt.vcf.gz} -c {sample_gt.vcf.gz} -o {bench_dir} --gtcomp -r 1000 -p 0.00
